# Prevalence and Factors Associated with Apparent Treatment-Resistant Hypertension Among Adults with Hypertension Attending the Medical Outpatient Clinic at Mbarara Regional Referral Hospital: A Cross-sectional Study

**DOI:** 10.21203/rs.3.rs-7666726/v1

**Published:** 2025-09-23

**Authors:** Christopher Mugabo, Andrew Mutekanga, Josephine Najjuma, Martha Sajatovic, Mark Kaddumukasa, Elly Katabira, Keneth Kananura, Fardous Abeya

**Affiliations:** Mbarara University of Science and Technology; Mbarara University of Science and Technology; Mbarara University of Science and Technology; Case Western Reserve University School of Medicine; Makerere University College of Health Sciences; Makerere University College of Health Sciences; Mbarara Regional Referral Hospital; Mbarara University of Science and Technology

**Keywords:** Hypertension, Apparent Treatment-Resistant Hypertension, Mbarara Regional Referral Hospital

## Abstract

**Background:**

Apparent treatment-resistant hypertension (aTRH) poses a clinical challenge and is associated with poor cardiorenal outcomes. Despite the growing burden of hypertension in sub-Saharan Africa, data on aTRH remain scarce, particularly in Uganda. We sought to determine the prevalence and factors associated with aTRH among adults with hypertension.

**Methods:**

A cross-sectional study was conducted at MRRH, including patients with treated hypertension for ≥ 6 months, who had taken antihypertensives in the past 2 weeks. Apparent TRH was defined as office systolic blood pressure ≥140mmHg or diastolic blood pressure ≥ 90mmHg in a patient on ≥3 antihypertensive agents (including a diuretic) or ≥4 antihypertensive agents irrespective of blood pressure level. Simple proportions were used for the prevalence of aTRH, while modified Poisson regression with robust variance was used for associated factors, reporting adjusted prevalence ratios (aPR) with 95% confidence intervals (CI).

**Results:**

A total of 250 participants were enrolled. The mean age was 58 (±13) years, and 200 (80%) were female. The prevalence of aTRH was 30.8% (95% CI: 25.4–36.8). Male aPR 1.90 (95% CI: 1.23–2.94; p= 0.004), eGFR <60 mL/min/1.73m^2^ aPR 1.81 (95% CI: 1.23–2.64; p=0.002), and increasing age aPR 0.98 (95% CI: 0.96–0.99; p= 0.003) were significantly associated with aTRH.

**Conclusion:**

Apparent TRH is highly prevalent, nearly one-third of treated patients, and is independently associated with male sex, impaired kidney function, and younger age. We recommend that targeted efforts focus on younger individuals, males, and those with impaired kidney function to improve blood pressure control and reduce associated risks.

## Introduction

According to the WHO, 1.4 billion people globally have hypertension, with the majority (2/3) living in limited-resource countries(1). The burden of hypertension is greatest in Africa (27%), and it has experienced the most substantial rise since 1990(2). Hypertension, especially when uncontrolled, is the most important modifiable determinant of premature cardiovascular deaths, which contributes the greatest (32%) to all deaths globally, with significant health and economic implications(3).

An estimated 30% of adults in sub-Saharan Africa are living with hypertension, with treatment rates at 18%, and control rates as low as 7%(4). Population studies in Uganda, and also Mbarara, estimate the prevalence of hypertension to be as high as 33% (5–8). Despite the efficacy of anti-hypertensive medications, only about one-fifth of patients with hypertension (globally), and 8% of those in Uganda are controlled(9).

Apparent treatment-resistant hypertension (aTRH) is a term used in epidemiological research to describe individuals with resistant hypertension but in whom at least one of the following data elements is missing: medication dose, adherence, or out-of-office blood pressure; thus, pseudo-resistance cannot be excluded (10, 11). It has been adopted by major guidelines due to the feasibility challenges associated with determining true resistant hypertension in a real-life setting. Patients with aTRH have a 2 to 3-fold higher risk of cardiorenal events and all-cause death compared to their responder-counterparts(12, 13).

The underlying mechanism of resistant hypertension is often unclear; however, studies suggest increased sodium and water retention, increased sympathetic activity, and enhanced renin-angiotensin-aldosterone system, which leads to arterial stiffness, myocardial fibrosis, and vascular remodeling(14). These form the backbone for the hypertension-mediated organ damage observed in patients with resistant hypertension

Despite the region having the highest burden of hypertension, there is limited data on aTRH in sub-Saharan Africa. This study sought to determine the burden of aTRH among patients with hypertension at Mbarara Regional Referral Hospital.

## Methodology

### Study Design and Setting

This was a hospital-based, cross-sectional study conducted at the hypertension clinic of Mbarara Regional Referral Hospital (MRRH). MRRH serves about five million people across several districts. The weekly clinic, supervised by specialist physicians, manages approximately 380 hypertension patients monthly.

### Sample Size and Sampling Technique

The sample size was calculated using the Kish & Leslie formula with an expected prevalence of apparent treatment-resistant hypertension (aTRH) at 18.9%, as determined in a Ghanaian study by Ayisi-Boateng et al(15). A minimum sample size of 235 was estimated, and adjusted for a 5% non-response rate, resulting in a target sample size of 247 participants. A total of 250 participants were ultimately enrolled using a consecutive sampling method.

### Eligibility Criteria

We included adults (aged ≥ 18 years) with treated hypertension for at least six months, who had taken antihypertensive medication within the prior two weeks, and provided written informed consent. We excluded patients with hypertensive crises requiring immediate stabilization.

### Study Procedures and Study Variables

Patients meeting eligibility criteria provided written informed consent. A trained study nurse and study investigator collected data on demographics, lifestyle behaviors (alcohol, smoking, diet, physical activity), comorbidities (including diabetes, HIV, chronic obstructive pulmonary disease/asthma), medication use, electrocardiographic, and echocardiographic images for left ventricular mass assessment, and blood draws for serum creatinine, low density lipoprotein and random blood glucose determination.

Alcohol use was assessed using the Alcohol Use Disorders Identification Test – Consumption (AUDIT-C), adapted for local alcohol equivalents, and physical activity was measured using the International Physical Activity Questionnaire – Short Form (IPAQ-SF), adapted for the local context.

### Clinical Measurements

Blood pressure was measured using a validated Sinocare AES-U181 device. Three readings were taken with a resting interval, and the final BP was computed as the average of the last two readings. Anthropometric measurements included weight, height, BMI, and neck circumference. ECGs were recorded using the PC ECG 2.1 SEMIP 1.5 system.

Echocardiograms were performed using a Sonosite M-Turbo machine with a phased-array transducer. Left ventricular mass was calculated from M-mode measurements and indexed to BSA using the Mosteller formula. Left ventricular hypertrophy was defined per ASE guidelines.

Venous blood was analyzed for serum creatinine and LDL cholesterol using the HumaStar 200 analyzer. Random blood glucose was measured with an On Call^®^ Plus glucometer using finger-prick samples.

### Statistical Analysis

Data were captured in REDCap and exported to STATA version 17. Categorical variables were presented as frequencies and percentages, and continuous variables as means (with standard deviations) or medians (with interquartile ranges). Prevalence of aTRH was calculated as a proportion. Modified Poisson regression with robust standard errors was used to estimate prevalence ratios (PRs) for factors associated with aTR reporting aPR with 95% CI. Variables with p < 0.05 or biological relevance were included in multivariable models. Significance was set at p < 0.05.

## Results

### Participant Recruitment and Baseline Characteristics

Between 10th September 2024 and 3rd April 2025, 300 patients attending the hypertension outpatient clinic at MRRH were approached. Of these, 275 met eligibility criteria, but 20 declined participations, and 5 had hypertensive crises requiring stabilization. Ultimately, 250 participants were enrolled

As in Table 1, the mean age was 58 ± 13 years, with females constituting 80% of the sample. Residence was nearly evenly split between rural (52.4%) and urban (47.6%) settings. The median distance to the hospital was 7 km (IQR 4–24). Most participants had attained primary education (56%), and 49.2% were married. Half of the participants were peasant farmers.

Alcohol consumption was reported by 12% of participants, and only one participant was a current smoker. Participants were generally physically active, with a median MET-min/week of 4893 (IQR 3312–7326). The median duration of hypertension was 4 years (IQR 2–10). Comorbidities included diabetes mellitus (11.6%), HIV (10.4%), and Chronic obstructive pulmonary disease/asthma (1.2%).

The mean systolic and diastolic blood pressures were 144 ± 23 mmHg and 89 ± 14 mmHg, respectively. Mean BMI was 29.3 ± 5.8 kg/m^2^, and mean mid-upper arm circumference was 31 ± 4 cm.

### Prevalence of Apparent Treatment-Resistant Hypertension

**Figure 1:**
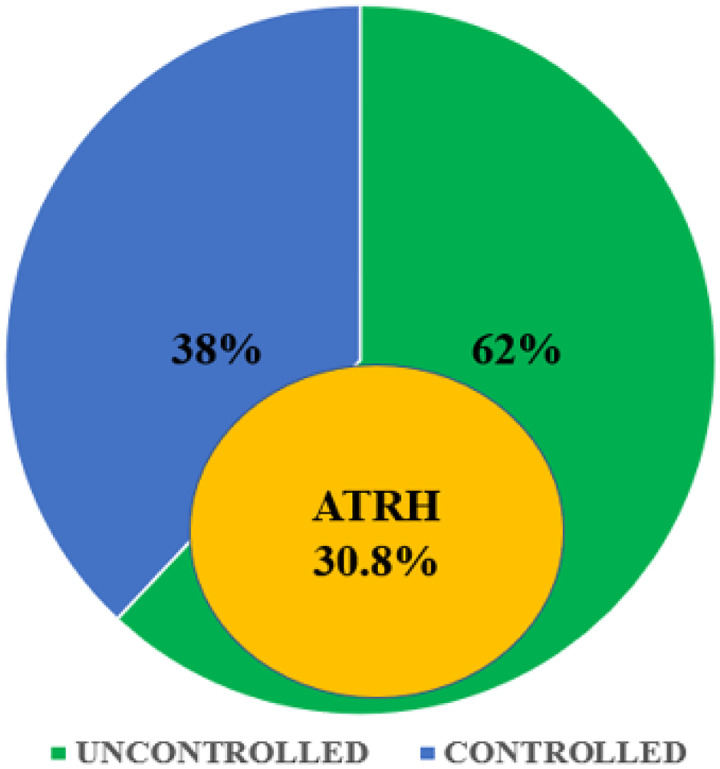
Blood Pressure Control and Prevalence of Apparent Treatment-Resistant Hypertension

Among the 250 participants, 155 (62%) had uncontrolled blood pressure (≥ 140/90 mmHg). The overall prevalence of apparent treatment-resistant hypertension was 30.8% (95% CI: 25.4–36.8), identified in 77 participants Fig. 1. Of these, 63 (81.8%) were on three antihypertensive drugs (including a diuretic), while 14 (18.2%) were on four or more antihypertensives.

### Factors Associated with aTRH

In multivariable analysis (Table 2), Male sex: aPR 1.90 (95% CI: 1.23–2.94; p = 0.004), eGFR < 60 ml/min/1.73m^2^: aPR 1.81 (95% CI: 1.23–2.64; p = 0.002), Increasing age (years): aPR 0.98 (95% CI: 0.96–0.99; p = 0.003) were found to be statistically significant. Although not statistically significant, trends toward higher prevalence of aTRH were observed among participants with current alcohol use, longer hypertension duration, obesity, and LDL cholesterol > 100 mg/dL.

## Discussion

In our study, we found that aTRH was highly prevalent at 30.8%, and was significantly associated with male sex, reduced glomerular filtration rate (eGFR < 60ml/min/1.73m2), and younger age.

The observed prevalence is high and underscores the burden of this high-risk phenotype of hypertension. The figure is relatively high compared to studies in other regions. A multicenter cross-sectional study from Ghana reported an aTRH prevalence of 18.9%(15). Of note, different centers in this study noted significant variability in the prevalence of aTRH ranging from 0.6% to 26.2%. A similar study in Ethiopia showed the prevalence of aTRH to be around 8.6%(17). One pooled cross-sectional analysis that included black-American participants from 2 large cohort studies (Jackson Heart Study and REGARDS), estimated a prevalence of 28.3%, closer to our observed aTRH prevalence(18). Our prevalence of aTRH is, however, lower than that determined among post-stroke patients in Ghana, which was reported to be 42.7%(19).

We hypothesize that this observed high prevalence of aTRH was contributed by, first, that our study was conducted in a tertiary care hospital, which typically manages more complex, or refractory cases, including patients referred due to uncontrolled hypertension. Secondly, the requirement to have taken anti-hypertensive medications in the past 2 weeks, although desired to identify patients who were on pharmacological therapy, could have contributed to the observed high prevalence of aTRH. By excluding those who were non-adherent or off treatment for longer durations, individuals likely resistant to therapy may have been selectively included. This is backed by the observation of a higher proportion of patients with controlled blood pressure (38%) in our study as opposed to the estimated 8% in Uganda, and 20% globally(9). Most population-based studies on aTRH in literature have often included all patients with a diagnosis of hypertension(12, 20, 21).

Male participants had 90% higher prevalence of aTRH compared to their female counterparts i.e aPR 1.90, p = 0.004. This aligns with previous research that shows that men are more likely to have RH(22–25). This is potentially due to biological and behavioral factors. Androgens are linked to the preferential blood pressure rise in men, by activating the renin-angiotensin-aldosterone system, which causes sodium reabsorption through angiotensin II or androgen-mediated increase in aldosterone(26). Men are also more likely to engage in lifestyle behaviour that negatively impacts blood pressure control e.g., poor dietary habits, low health-seeking behaviour, and higher alcohol and tobacco use. In our study, however, there was an equal proportion of females and males currently using alcohol.

A reduced estimated glomerular filtration rate (< 60ml/min/1.73m^2^) was independently associated with Atrh, aPR 1.81, p = 0.002. This is in tandem with previous evidence linking an eGFR < 60ml/min/1.73m^2^ with aTRH(15). The underlying mechanisms include increased salt and water retention, arterial stiffness, and excessive renin-angiotensin system activation in patients with kidney dysfunction.

Interestingly, increasing age was independently associated with a lower prevalence of aTRH. Specifically, each additional year of age was associated with a 2% decrease in the prevalence of aTRH, aPR = 0.98; p = 0.003. This is contrary to many prior studies that suggests that aTRH increases with age (13, 27, 28). Our results, however, align with findings from a transcontinental study investigating aTRH in stroke survivors, which also found that each year rise was associated with lower odds of aTRH i.e aOR 0.99 (95% CI 0.98–0.99, p < 0.0002). This finding might suggest that younger patients with resistant hypertension develop complications or die earlier, so that those who survive longer are those with an inherently lower risk for aTRH, and/or more likely to be adherent to medications. Furthermore, there is literature to suggest that cardiovascular complications occur a decade earlier among Africans compared to other individuals from other regions (29, 30).

Having current alcohol use, duration of hypertension, obesity, and a low-density lipoprotein cholesterol ≥ 100mg/dl, although not statistically significant, showed a trend to increased odds of aTRH. This is likely due to, among other reasons, type II error due to a relatively small sample size.

## Conclusion

Our study found the prevalence of aTRH to be high, 30.8% (nearly one-third of patients with hypertension), and was independently associated with male sex, reduced kidney function, and younger age.

### Recommendation

Based on our findings, we recommend that clinicians screen patients with hypertension for aTRH, especially if they are males and younger. We also recommend routine screening for kidney dysfunction in similar patient populations.

For future research, we recommend a prospective cohort study integrating ambulatory or home-based blood pressure monitoring and objective medication adherence monitoring to more precisely determine the incidence and predictors of true resistant hypertension in our setting.

### Strengths

To our knowledge, this is the first study conducted in Uganda on apparent treatment-resistant hypertension (aTRH), thereby contributing valuable data to the limited body of literature on this important topic. The study population was relatively homogeneous, with a low burden of comorbidities, which enhances the generalizability of the findings to individuals with hypertension only.

### Limitations

The study has limitations, including its cross-sectional design, which limits the ability to infer causality, and the singlecenter nature of the study may restrict the generalizability of the findings. The inclusion of patients who had taken antihypertensives in the past 2 weeks, though desired, may have introduced a selection bias, ultimately inflating the observed prevalence of aTRH.

## Figures and Tables

**Table 1:** Baseline characteristics of study participants attending the medical outpatient department at MRRH

Variable	N = 250
Age in years, mean (± SD)	58 (± 13)
Female sex, n (%)	200 (80.0)
Residence type, n (%) Rural	131 (52.4)
Urban	119 (47.6)
Education level, n (%) None	40 (16.8)
Primary	140 (56.0)
Secondary/Tertiary	70 (28.0)
Marital status = n (%) Married	123 (49.2)
Single/Never married	20 (8.0)
Separated/Widowed/Divorced	107 (42.8)
Peasant farmers, n (%)	95 (50.3)
Alcohol use (Current), n (%)	31(12.4)
Physical activity (MET-min/week), Low < 600	5 (2)
Moderate 600–3,000	53 (21.2)
High > 3,000	192 (76.8)
Duration of hypertension (years), median (IQR)	4 (2–10)
Comorbidities, n (%) Diabetes mellitus	29 (11.6)
HIV	26 (10.4)
Anti-hypertensive medications, n (%) ARB/ACEi	190 (76.0)
Diuretics	184 (73.6)
CCBs	176 (70.4)
At least 3 anti-hypertensives (Including a diuretic)	104 (41.6)
At least 4 anti-hypertensives	14 (5.6)
**Clinical measurements**, mean (± SD) SBP (mmHg)	144.5 (± 22.7)
DBP (mmHg)	88.7 (± 14.2)
BMI, kg/m^2^, mean (± SD)	29.3 (± 5.8)
MUAC (cm), mean (± SD)	31 (± 4)
Left ventricular hypertrophy, n (%) N = 248	99 (40.0)
**Laboratory measurements**	
eGFR (ml/min/1.73m^2^), mean (± SD)	63.2 (± 18)
Low-density lipoprotein cholesterol (mg/dl), mean (± SD)	129.6 (± 46.7)
Random blood glucose (mmol/l), median (IQR)	5.6 (4.9–6.9)

MET Metabolic Equivalent of Task; HIV Human Immunodeficiency Virus; ARB Angiotensin II Receptor Blocker; ACEi Angiotensin-Converting Enzyme Inhibitor; CCB Calcium Channel Blocker; MMAS-U 8-item Morisky Medication Adherence Scale (Uganda); SBP Systolic Blood Pressure; DBP Diastolic Blood Pressure; BMI Body Mass Index; MUAC Mid-Upper Arm Circumference; eGFR Estimated Glomerular Filtration Rate; SD Standard Deviation; IQR Interquartile Range

**Table 2:** Factors associated with apparent treatment-resistant hypertension in univariate and multivariable analyses

Variable	Unadjusted Prevalence Ratio (PR)	95% Confidence Interval (CI)	P-value	Adjusted Prevalence Ratio (aPR)	95% Confidence Interval (CI)	P-value
Age (Each 1-year increase)	0.99	0.98– 1.00	0.160	**0.98**	**0.96–0.99**	**0.003**
Sex (Male) *(Ref. Female)*	**1.70**	**1.17–2.49**	**0.006**	**1.90**	**1.23–2.94**	**0.004**
Residence (Urban) *(Ref. Rural)*	1.21	0.83–1.77	0.320	-	-	-
Alcohol use (Current) *(Ref. No alcohol use)*	**2.00**	**1.36–2.95**	**0.000**	1.44	0.97–2.14	0.067
Physical activity (< 3000 MET-min/week) *(Ref. ≥3000 MET-min/week)*	1.01	0.65–1.57	0.965	1.04	0.68–1.61	0.851
Duration of hypertension (≥ 5years) *(Ref. <5 years)*	1.40	0.96–2.04	0.082	1.45	0.99–2.12	0.053
DM	0.88	0.47–1.65	0.697	0.88	0.48–1.63	0.686
HIV	0.60	0.27–1.35	0.215	-	-	-
Obesity, BMI ≥ 30 kg/m^2^ *(Ref. <30kg/m^2^)*	1.19	0.82–1.73	0.361	1.45	0.98–2.14	0.065
eGFR, < 60 ml/min/1.73m^2^ *(Ref. ≥60ml/min/1.73m^2^)*	**1.67**	**1.15–2.43**	**0.008**	**1.81**	**1.23–2.64**	**0.002**
LDL cholesterol (≥ 100mg/dl) *(Ref. <100mg/dl)*	1.42	0.86–2.35	0.170	1.52	0.94–2.44	0.086

HIV human immunodeficiency virus, DM diabetes mellitus, BMI body mass index, eGFR estimated glomerular filtration rate, LDLc low-Density Lipoprotein Cholesterol

## Data Availability

The datasets used during this study are available from the corresponding author upon reasonable request.

## Abbreviations

aTRH: Apparent Treatment–Resistant Hypertension
BMI: Body Mass Index
CI: Confidence Interval
IQR: Interquartile Range
MET: Metabolic Equivalent Task
MRRH: Mbarara Regional Referral Hospital
PR: Prevalence Ratio
